# Specialty preferences and influencing factors among undergraduate medical students in Ain Shams University

**DOI:** 10.1186/s12909-026-08603-2

**Published:** 2026-02-10

**Authors:** Yosra S. Abd El-Ghaffar, Abeer Abd El Salam

**Affiliations:** Community, Environmental, and Occupational Medicine Department, Faculty of Medicine, Ain Shams University, Cairo, Egypt

**Keywords:** Medical specialty preference, Medical students, Determining factors, Clinical specialties, Egypt, Career choice

## Abstract

**Background:**

Selecting a medical specialty is a vital step for medical students. Numerous cultural, educational, and personal variables impact this decision. The purpose of this study is to determine the distribution of specialty choices among medical students at Ain Shams University, to identify factors that influence these preferences, and to compare specialty preferences and influencing factors between Egyptian and non-Egyptian students.

**Methods:**

About 413 medical students of Ain Shams University participated in a cross-sectional study, comprising both Egyptian and non-Egyptian students across all academic years and internship levels. Stratified random sampling was performed. Data was gathered via a standardized, self-administered questionnaire including demographic and social data, specialty preferences, and reasons for those preferences.

**Results:**

Clinical specializations were selected by more than 94% of students, with internal medicine and general surgery coming in first (each at 12.3%), next being gynecology and obstetrics (11.6%). Passion and personal interest were the top drivers of specialty choice (69.2%), consistent across genders and nationalities. Male students showed a modest preference for specialties traditionally associated with higher income potential, whereas female students were more likely to express interest in specialties emphasizing patient-centered care. Over half (51.6%) expressed a preference to work abroad, particularly among non-Egyptian students.

**Conclusion:**

Most medical students preferred clinical specialties, with personal passion being the strongest motivator. Nationality and gender significantly affect both specialty choice and future work location. These results emphasize the necessity of laws that deal with both student aspirations and the broader needs of the healthcare system.

**Supplementary Information:**

The online version contains supplementary material available at 10.1186/s12909-026-08603-2.

## Introduction

Medical school graduates form the foundation of a country’s future healthcare workforce, making the selection of medical specialties a critical determinant of national health system performance. Achieving a balanced distribution of physicians across disciplines requires understanding how graduates formulate their specialty preferences and the factors that guide these decisions [1]. Concentration in a limited number of popular fields may contribute to shortages in essential specialties, inequitable access to healthcare, regional disparities in service availability, and increased healthcare costs [2–5]. International studies have shown that students’ decisions are influenced by personal interest, anticipated job satisfaction, perceived fit with personality, academic and intellectual considerations, lifestyle aspirations, and cultural expectations. Evidence from Saudi Arabia, China, and the United States demonstrates that lifestyle considerations, sociocultural norms, and exposure during training shape the prioritization of clinical over research careers [4, 6–9].

Despite the extensive global literature, limited national data exist regarding the determinants of specialty choice in Egypt. Beyond individual preferences, structural and policy-level factors also shape career trajectories. Postgraduate training in Egypt typically requires passing competitive national examinations to access residency positions, with centralized allocation of unfilled places. Furthermore, the country faces substantial physician emigration, with tens of thousands of Egyptian physicians working abroad and increasing resignation rates from governmental hospitals, exerting pressure on the domestic workforce [10–12]. Within this context, “better choices” reflect alignment between students’ interests, competencies, and available opportunities, taking into account structural factors such as exam requirements, and workforce needs. While a “better future” refers to increased career satisfaction, reduced mismatch between preference and available training slots, and improved retention within the national health system [13, 14].

Medical specialty imbalances are a growing global concern, with many countries experiencing persistent shortages in fields such as primary care, psychiatry, and public health—challenges that threaten the equity and resilience of health systems [15, 16]. Against this backdrop, there is a notable scarcity of empirical evidence in Egypt regarding how medical students articulate their specialty preferences and how contextual, policy, and sociocultural factors influence these decisions. This study therefore aims to find out what percentage of medical students at Ain Shams University’s Faculty of Medicine are interested in each different specialty, identify what factors and reasons influence these students’ specialty preferences, and to compare the Egyptian and non-Egyptian students in terms of which specialties they prefer and what influences their choices.

## Methods

A cross-sectional study was carried out to achieve the study objectives.

### Study population and setting

The study was carried out at the Faculty of Medicine, Ain Shams University (ASU), Cairo, Egypt. ASU is one of the largest medical schools in the Middle East and enrolls both Egyptian and non-Egyptian students within a unified medical curriculum.

The study population comprised medical students from all stages of undergraduate medical education, including:

- Pre-clinical (basic science) years (Years 1–3),
- Clinical years (Years 4–5), and
- Internship training years (first and second year of internship).

Although definitive specialty decisions are often made during the clinical years or internship, evidence suggests that early career preferences may emerge during the pre-clinical phase and influence later choices. Including students across all academic stages allowed for the assessment of both early inclinations and more established preferences, providing a comprehensive view of factors shaping career decision-making and informing future curriculum planning and career guidance strategies [17].

Given the diversity of the student body in terms of nationality and cultural background, nationality was included as a key variable to explore its potential influence on specialty preference patterns.

### Eligibility criteria

Inclusion criteria:

- □ Medical students (male and female) enrolled at Ain Shams University from the first to the fifth academic year who provided informed consent. 
- □ Medical interns in their first or second year of training who provided informed consent, whether rotating at El-Demerdash University Hospital or Ministry of Health and Population hospitals.

#### Study context

This study was conducted at the Faculty of Medicine, Ain Shams University (ASU), Cairo, Egypt. Ain Shams University is one of the oldest and largest public universities in the Middle East and North Africa region. The Faculty of Medicine offers a seven-year medical education program, consisting of three pre-clinical (basic science) years, two clinical years, followed by two compulsory internship years. The curriculum is standardized across all students and follows nationally regulated medical education requirements.

The faculty enrolls a heterogeneous student population, including both Egyptian and non-Egyptian students from diverse cultural and socioeconomic backgrounds, all trained within the same educational and clinical framework. Clinical training is primarily delivered at El-Demerdash University Hospital, a large tertiary-care teaching hospital, as well as affiliated Ministry of Health and Population hospitals during the internship period. This diverse and unified educational setting provides an appropriate context for examining factors influencing medical specialty preferences.

Therefore, nationality was included to assess whether diversity influences specialty preference trends.

#### Study period

Data was collected within 4 months.

#### Sample size

Using Epi Info 7 program for sample size calculation, reviewing results from a previous relevant study [18] showed that the most preferable specialty among medical students was internal medicine (19.8%), with a margin of error = 7% design effect = 2, and at a 95% confidence level, a sample size of 250 students (125 Egyptian and 125 non-Egyptian) was needed to achieve the study objectives.

#### Sampling method

In this study, we used a stratified random sampling method to choose participants from the student population. To ensure adequate representation and allow for meaningful subgroup analysis, the sample was stratified based on nationality. Two distinct strata were defined: Egyptian students and non-Egyptian students. Participants were chosen at random from each stratum based on how many people were in that stratum overall. This method was chosen to minimize sampling bias and get a fair representation of all students, thereby increasing the study’s findings validity and reliability.

#### Study tools

A questionnaire in the English language was designed after reviewing the literature to be self-completed by participants fulfilling the inclusion criteria. The questionnaire was adapted from previously validated instruments used in studies of medical specialty preference, with modifications to suit the local context. Its content validity was assessed through review by three experts in medical education and public health. A pilot test was conducted with 30 students to ensure clarity and feasibility [3, 4, 18–20].

Three sections made up the questionnaire: The first section contains the students’ sociodemographic information, including their age, sex, nationality, parents’ level of education, and occupation. In the second section, students were given a choice of specialties and asked to select their desired postgraduate field, which included both clinical and non-clinical specialties. Specialties were categorized according to internationally recognized groupings. Clinical specialties involve hospital-based specialties (e.g., internal medicine, general surgery, pediatrics), primary care specialties (e.g., family medicine), and diagnostic/support specialties (e.g., radiology, pathology, anesthesia). Non-clinical career pathways involve alternative professional tracks such as public health, biomedical research, and medical education. Clinical specialties also can be classified into surgical specialties—such as general surgery, orthopedics, and neurosurgery—which involve operative procedures and higher technical demands, and non-surgical specialties—such as internal medicine, pediatrics, and psychiatry—which emphasize diagnostic evaluation and longitudinal patient care. In the third section, students were asked questions to investigate the factors influencing their preferences for specialization. (Supplementary file 1)

Study participants were required to specify which contributing factors they thought affected choosing their specialization. The predetermined parameters reflect the students’ personal preferences regarding their future careers, their preferred medical specialty features, and the social and financial factors affecting their choices.

About 30 students were used in a pilot study to test the questionnaire’s clarity and feasibility, with these participants subsequently excluded from final analysis. Required modifications were implemented: redundant questions were identified and eliminated, while ambiguous items were clarified.

#### Data management and statistical analysis

The gathered data were examined for accuracy and completeness, then systematically coded. Data analysis was performed according to variable types using SPSS version 27.0. The mean ± standard deviation was used to display the numerical data. Frequency tables and percentages were used to depict categorical data, while chi-square tests were used to assess the relationships between categorical variables. Logistic regression was used to evaluate the independent impact of each motivational factor for specialty preference. When *p* ≤ 0.05, the results were deemed statistically significant.

## Results

Table 1 illustrates sociodemographic characteristics of 413 participating students. The average age of the participants was 21.27 ± 2.35 years. Female students comprised a slightly larger portion of the sample (54.5%) than male students (45.5%). Nearly all students are single (97.8%). The sample included students across all years, with a larger proportion of 4th-year students (30.5%) reflecting enrollment and availability at the time of data collection, followed by second-year students (21.1%) and interns (12.8%). Moreover, 49.9% of the students have a CGPA between 3% and 3.5%. Nearly equal percentages of Egyptian (48.9%) and non-Egyptian (51.1%) students participated, indicating a diverse international student organization. A high proportion of fathers (47.7%) and mothers (47.2%) have a college degree, with a significant portion also holding postgraduate qualifications (30.8% for fathers and 21.5% for mothers).

**Table 1:** Sociodemographic characteristics of the students (*n* = 413)

Sociodemographic characteristics	*N*	%
Age (mean ± SD) years	21.27 ± 2.35	
Gender		
Male	188	45.5
Female	225	54.5
Marital status		
Single	404	97.8
Married	9	2.2
Educational Level		
1 st year	60	14.5
2nd year	87	21.1
3rd year	61	14.8
4th year	126	30.5
5th year	26	6.3
Interns	53	12.8
Cumulative Grade Point Average (CGPA)		
2–2.4.4	85	20.6
2.5–2.9	122	29.5
3–3.5.5	206	49.9
Nationality		
Egyptian	202	48.9
Non-Egyptian	211	51.1
Father’s education level		
Primary	18	4.4
Intermediate	20	4.8
Secondary	51	12.3
College	197	47.7
Postgraduate studies	127	30.8
Mother’s education level		
Primary	32	7.7
Intermediate	18	4.4
Secondary	79	19.1
College	195	47.2
Postgraduate studies	89	21.5
Father’s job		
Retired	43	10.4
Medical and health field	66	16.0
Engineering field	53	12.8
Military field	13	3.1
Education field	57	13.8
A government employee	63	15.3
Free business Private sector	112	27.1
Dead	6	1.5
Mother’s job		
Housewife	198	47.9
Medical and health field	49	11.9
Engineering field	8	1.9
Education field	107	25.9
A government employee	22	5.3
Free business Private sector	28	6.8
Dead	1	0.2
A family relative working in the medical field		
Parents in medical field	62	15%
Siblings in medical field	82	19.9%
Other relatives in medical field	60	14.5%
No family member in medical field	209	50.6%

Fathers are mostly involved in the private sector (27.1%) and government jobs (15.3%), with a notable presence in the medical and engineering fields. Mothers are predominantly housewives (47.9%), but a considerable number are employed in education (25.9%) and healthcare (11.9%). Nearly half of the students (49.4%) have some form of familial connection to the medical field.

**Fig. 1:**
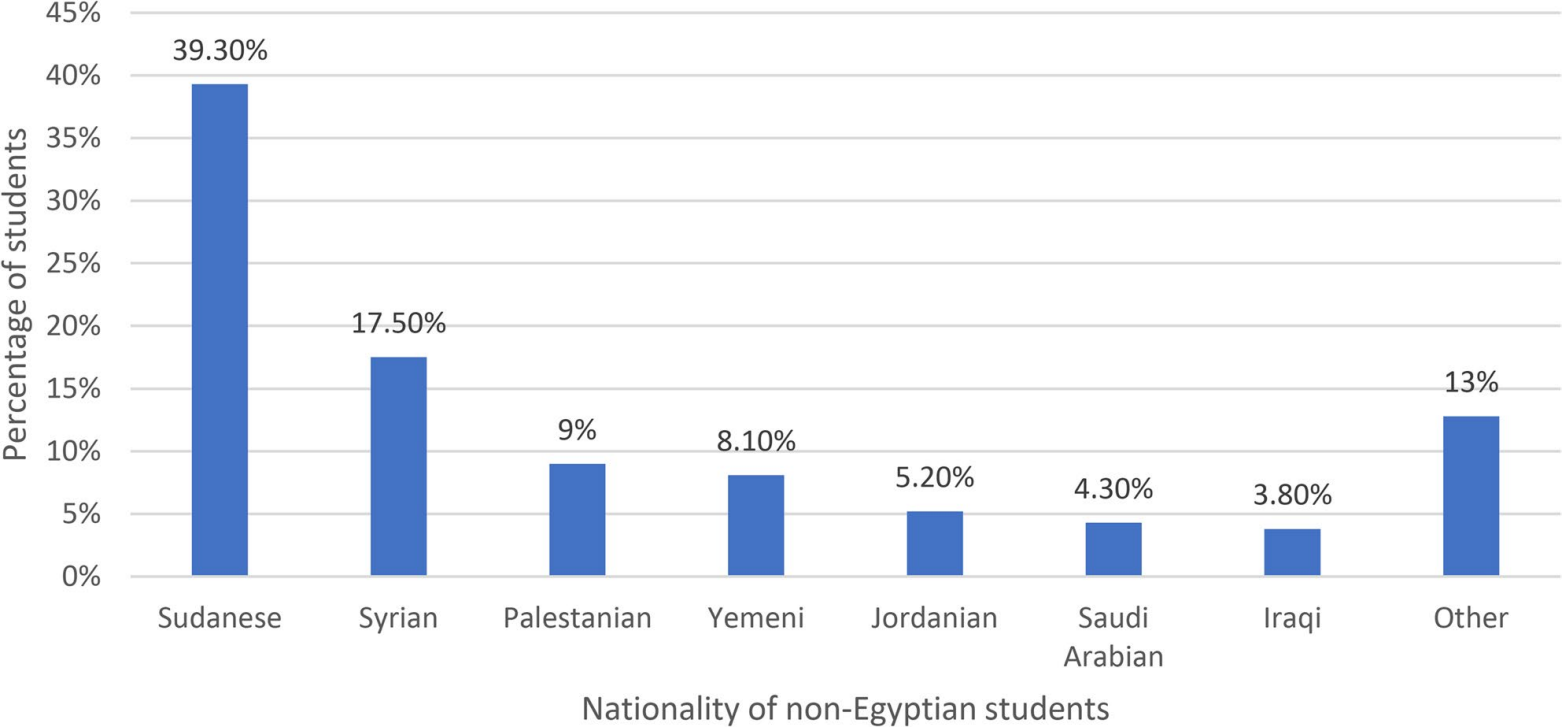
Nationality of the non-Egyptian students (*n* = 211). *Other include Somalian, Nigerien, Indian, Chadian, Afghan, Libyan, Emirati, Djiboutian, Mauritanian, Tunisian

Figure 1 illustrates the distribution of non-Egyptian students by nationality. Sudanese students constitute the largest group, with 39.3% of non-Egyptian students, followed by Syrians at 17.5%. Other remarkable groups include Palestinians (9%), Yemenis (8.1%), Jordanians (5.2%), Saudi Arabians (4.3%), and Iraqis (3.8%), with students from other nationalities, including Somalian, Indian, Nigerian, and others, collectively accounting for 13%.

According to Table 2, it is observed that a large percentage of pupils, irrespective of gender, nationality, or educational level, prefer clinical specialties, across all groups exceeding 94% in this choice. Non-clinical specialties are still considerably less popular, with only 4–5% of students choosing to identify as such, accompanied by a very small percentage remaining undecided. Gender, nationality, and academic year showed no statistically significant associations with specialty preference, which suggests that there is widespread consensus among students toward clinical specialties.

**Table 2:** Categories of the specialty chosen by gender, nationality, and educational level among the students

Category of the specialty		Clinical	Non clinical	Not decided	Total	X^2^	*P* value
		N (%)	N (%)	N (%)	N (%)		
Total		390 (94.4%)	17 (4.1%)	6 (1.5%)	413 (100.0%)	0.068	0.97
Gender	Male	177 (94.1%)	8 (4.3%)	3 (1.6%)	188 (100.0%)		
	Female	213 (94.7%)	9 (4.0%)	3 (1.3%)	225 (100.0%)		
Nationality	Egyptian	190 (94.1%)	10 (5.0%)	2 (1.0%)	202 (100.0%)	1.26	0.53
	Non-Egyptian	200 (94.8%)	7 (3.3%)	4 (1.9%)	211 (100.0%)		
Educational Level	1 st year	56 (93.3%)	3 (5.0%)	1 (1.7%)	60 (100.0%)	6.13	0.8
	2nd year	82 (94.3%)	5 (5.7%)	0 (0.0%)	87 (100.0%)		
	3rd year	58 (95.1%)	2 (3.3%)	1 (1.6%)	61 (100.0%)		
	4th year	118 (93.7%)	4 (3.2%)	4 (3.22%)	126 (100.0%)		
	5th year	25 (96.2%)	1 (3.8%)	0 (0.0%)	26 (100.0%)		
	interns	51 (96.2%)	2 (3.8%)	0 (0.0%)	53 (100.0%)		

Concerning specialty selection, Table 3 shows that gender and nationality significantly affect medical specialty preferences among the 413 students. Internal Medicine and General Surgery were the most common choices. Both Internal Medicine and General Surgery were selected by 12.3% of students each, with Obstetrics and Gynecology following at 11.6%. Male students primarily favored Internal Medicine and General Surgery, while female students demonstrated a strong preference for Obstetrics and Gynecology. Egyptian students showed preference towards obstetrics and gynecology, while non-Egyptian students leaned towards general surgery and cardiology. There were statistically significant differences based on gender (*p* < 0.001) and nationality (*p* = 0.014).

**Table 3:** Choice of the specialty by gender and nationality among the students (*n* = 413)

The specialty	Total	Egyptian	Non-Egyptian	Male	Female
	*N* (%)	*N* (%)	*N* (%)	*N* (%)	*N* (%)
Internal Medicine	51 (12.3%)	27 (13.4%)	24 (11.4%)	32 (17.0%)	19 (8.4%)
General Surgery	51 (12.3%)	21 (10.4%)	30 (14.2%)	29 (15.4%)	22 (9.8%)
Obstetrics and gynecology	48 (11.6%)	33 (16.3%)	15 (7.1%)	6 (3.2%)	42 (18.7%)
Cardiology or cardiac Surgery	47 (11.4%)	20 (9.9%)	27 (12.8%)	21 (11.2%)	26 (11.6%)
Neurology or neurosurgery	45 (10.9%)	22 (10.9%)	23 (10.9%)	20 (10.6%)	25 (11.1%)
Orthopedic	24 (5.8%)	12 (5.9%)	12 (5.7%)	19 (10.1%)	5 (2.2%)
Dermatology	19 (4.6%)	9 (4.5%)	10 (4.7%)	3 (1.6%)	16 (7.1%)
Pediatrics	19 (4.6%)	11 (5.4%)	8 (3.8%)	6 (3.2%)	13 (5.8%)
Non-clinical Specialty	17 (4.1%)	10 (5.0%)	7 (3.3%)	8 (4.3%)	9 (4.0%)
Psychiatry	15 (3.6%)	3 (1.5%)	12 (5.7%)	7 (3.7%)	8 (3.6%)
Ophthalmology	13 (3.1%)	7 (3.5%)	6 (2.8%)	6 (3.2%)	7 (3.1%)
Emergency Medicine	10 (2.4%)	1 (0.5%)	9 (4.3%)	2 (1.1%)	8 (3.6%)
Oncology	10 (2.4%)	3 (1.5%)	7 (3.3%)	6 (3.2%)	4 (1.8%)
Plastic Surgery	9 (2.2%)	7 (3.5%)	2 (0.9%)	8 (4.3%)	1 (0.4%)
Anesthesia	3 (0.7%)	3 (1.5%)	0 (0.0%)	1 (0.5%)	2 (0.9%)
Undetermined	6 (1.5%)	2 (1.0%)	4 (1.8%)	3 (1.6%)	3 (1.3%)
Other^@^	26 (6.3%)	11 (5.4%)	15 (7.1%)	11 (5.9%)	15 (6.7%)
Total	413 (100%)	202 (100%)	211 (100%)	188 (100%)	225 (100%)
*X*^*2*^		32.33		59.76	
*P* value		0.014*		0.000*	

^@^ Radiology, family medicine, urology, ENT, infection control, clinical pathology

*Statistically significant at *p* ≤ 0.05

Table 4 shows that the primary reason students select a medical specialty is passion for the subject and personal desire (69.2%), with no significant differences by gender or nationality. However, nationality showed a stronger influence than gender on other factors. There was a noteworthy statistical difference between Egyptian and non-Egyptian students regarding selecting their specialty based on the availability of job opportunities abroad (*p* = 0.049), the desire for increased personal or family time (*p* = 0.028), and minimal physical exertion (*p* = 0.004). Although gender differences were not statistically significant, there was a tendency for males to be more inclined to potential financial earnings, whereas females showed a slight preference for roles that involve helping others.

**Table 4:** Reasons behind specialty selection by nationality and gender (*n* = 413)

Reason	Total (*n* = 413)	Egyptian (*n* = 202)	Non-Egyptian (*n* = 211)	χ²	*P* value	Male (*n* = 188)	Female (*n* = 225)	χ²	*P* value
Passion for the subject & personal desire	286 (69.2%)	134 (66.3%)	152 (72.0%)	1.58	0.21	126 (67.0%)	160 (71.1%)	0.8	0.37
Expected high financial income	164 (39.7%)	87 (43.1%)	77 (36.5%)	1.86	0.17	84 (44.7%)	80 (35.6%)	3.56	0.06
High job opportunities abroad	103 (24.9%)	59 (29.2%)	44 (20.9%)	3.85	0.049*	54 (28.7%)	49 (21.8%)	2.64	0.1
More personal/family time	101 (24.5%)	59 (29.2%)	42 (19.9%)	4.83	0.028*	42 (22.3%)	59 (26.2%)	0.84	0.36
Influence from role model/teacher	86 (20.8%)	46 (22.8%)	40 (19.0%)	0.91	0.34	42 (22.3%)	44 (19.6%)	0.48	0.49
Innovative field/research opportunities	59 (14.3%)	29 (14.4%)	30 (14.2%)	0.002	0.97	23 (12.2%)	36 (16.0%)	1.19	0.28
Low physical exertion	57 (13.8%)	38 (18.8%)	19 (9.0%)	8.34	0.004*	28 (14.9%)	29 (12.9%)	0.35	0.56
Foreign scholarships	51 (12.3%)	30 (14.9%)	21 (10.0%)	2.29	0.13	25 (13.3%)	26 (11.6%)	0.29	0.59
Desire to help people	40 (9.7%)	20 (9.9%)	20 (9.5%)	0.021	0.508	13 (6.9%)	27 (12%)	3.03	0.057
Advised by parents/friends	29 (7.0%)	14 (6.9%)	15 (7.1%)	0.005	0.94	11 (5.9%)	18 (8.0%)	0.72	0.39
Prefer teaching/academia	7 (1.7%)	5 (2.5%)	2 (0.9%)	—	0.28#	2 (1.1%)	5 (2.2%)	—	0.46 #
Severe shortage in area	3 (0.7%)	1 (0.5%)	2 (0.9%)	—	1.0 #	3 (1.6%)	0 (0.0%)	—	0.09 #
Hard-to-reach area	1 (0.2%)	0 (0.0%)	1 (0.5%)	—	1.0 #	1 (0.5%)	0 (0.0%)	—	0.46 #
No interaction/self-reliance	1 (0.2%)	1 (0.5%)	0 (0.0%)	—	0.49#	1 (0.5%)	0 (0.0%)	—	0.46 #

* Statistically significant at *P* ≤ 0.05

# (Fisher Exact)

**Table 5:** Choice determinants associated with specialty preference

	OR	*P*
Surgical specialties		
Expected high financial income.	2.126 (1.345–3.361)	0.001
Can dedicate more time to myself and my family.	0.445 (0.250–0.790.250.790)	0.006
Innovative field in medicine/great opportunity for scientific research in this specialty.	0.334 (0.176 − 0.636)	0.001
Influenced by an ideal or a role model or a teacher	2.017 (1.171–3.474)	0.011
Non-surgical specialties		
Expected high financial income.	0.508 (0.322–0.802.322.802)	0.004
Can dedicate more time to myself and my family.	1.837 (1.050–3.214)	0.033
Innovative field in medicine/great opportunity for scientific research in this specialty.	2.539 (1.377–4.682)	0.003
Influenced by an ideal or a role model or a teacher	0.477 (0.275–0.825.275.825)	0.008
Non-clinical specialties		
I prefer to work in teaching and academic institutions only.	10.348 (1.508–71.006)	0.017

This logistic regression analysis in Table 5 reveals distinct motivational patterns driving specialty selection among medical students. Financial expectations strongly predict surgical career choice (OR = 2.126, *p* = 0.001) while showing a protective effect against non-surgical specialties (OR = 0.508, *p* = 0.004). Work-life balance considerations steer students away from surgery (OR = 0.445, *p* = 0.006) and toward non-surgical fields (OR = 1.837, *p* = 0.033). Research opportunities attract students to non-surgical specialties (OR = 2.539, *p* = 0.003) more than surgical ones (OR = 0.334, *p* = 0.001). Notably, mentorship shows the strongest influence on academic career pursuit (OR = 10.348, *p* = 0.017).

The sensitivity analyses demonstrated that the direction and magnitude of associations between the major predictors (e.g., great opportunity for scientific research, lifestyle factors, financial considerations) and specialty preference remained consistent across all alternative models. Excluding outliers (undecided students) or restricting the sample to clinical-year students did not materially alter the findings. All significant predictors in the primary model remained significant in the sensitivity models, supporting the robustness of the results.

Table 6 demonstrates the place where students prefer to work in the future. The distribution of students’ preferred future practice settings reflected the principal employment pathways recognized by the Egyptian Ministry of Health. Accordingly, the options provided in the questionnaire—public (government) hospitals, university teaching hospitals, private-sector institutions, military-affiliated healthcare facilities, and opportunities to work abroad—were selected to align with nationally established career tracks and to ensure that responses corresponded to the actual employment routes available to graduates.

**Table 6:** Place where the students prefer to work by nationality and gender

The place	Total (*n* = 413)	Egyptian (*n* = 202)	Non-Egyptian (*n* = 211)	Male (*n* = 188)	Female (*n* = 225)
	*N* (%)	*N* (%)	*N* (%)	*N* (%)	*N* (%)
Settle abroad	213 (51.6%)	91 (45.0%)	122 (57.8%)	101 (53.7%)	112 (49.8%)
Private sector	65 (15.7%)	39 (19.3%)	26 (12.3%)	27 (14.4%)	38 (16.9%)
Government (public) hospitals	57 (13.8%)	19 (9.4%)	38 (18%)	22 (11.7%)	35 (15.6%)
Teaching (university) hospitals	48 (11.6%)	39 (19.3%)	9 (4.2%)	22 (11.7%)	26 (11.6%)
Armed forces hospitals	11 (2.7%)	6 (3.0%)	5 (2.4%)	5 (2.7%)	6 (2.7%)
Medical research	12 (2.9%)	4 (2.0%)	8 (3.8%)	5 (2.7%)	7 (3.1%)
Settle in rural areas	7 (1.7%)	4 (2.0%)	3 (1.4%)	5 (2.7%)	2 (0.9%)
undetermined	5 (1.2%)	4 (2.0%)	1 (0.5%)	3 (1.6%)	2 (0.9%)
X^2^		38.36		4.24	
*P* value		0.00*		0.84	

*Statistically significant at *p* ≤ 0.05

The results demonstrate that over half of the students (51.6%) expressed a preference to settle abroad, being more among non-Egyptians (57.8%) than Egyptians (45.0%). Nationality plays a significant role in determining preferred work locations (*P* = 0.00), as Egyptians are more inclined to choose the private sector (19.3%) and teaching hospitals (19.3%), whereas non-Egyptians tend to prefer governmental hospitals (17.5%) and medical research (3.8%). However, regarding these preferences, no statistically significant differences between males and females were found (*P* = 0.84), since males and females showed similar preferences across all categories. Overall, the results show a high preference for working abroad and significant differences based on nationality but not gender.

## Discussion

This study provides a comprehensive assessment of the medical student population (*n* = 413), primarily of Arab origin, focusing on their preferred medical specialties, reasons for choosing these fields, and the desired future career path. The data collected from the region has been supplemented with existing literature to illustrate how diverse global findings align with local patterns and highlight prominent intersections.

The present findings contribute to the international literature by demonstrating how specialty preferences are shaped simultaneously by individual interests and broader structural determinants, including perceived cultural expectations, national health-system priorities, and perceived career opportunities. The comparison between Egyptian and non-Egyptian students highlights that even within a shared institutional setting, variations in cultural background and exposure to different healthcare systems substantially influence specialty aspirations—supporting previous work showing that national context is a major determinant of medical workforce patterns [21, 22]. Given that Ain Shams University trains a large and heterogeneous student body, the results may have relevance for other medical schools in multicultural regions or those hosting sizeable cohorts of international students. By situating specialty choice within both personal and systemic frameworks, the study enhances the generalizability of its findings and underscores the importance of considering national and cultural influences when developing policies aimed at addressing specialty shortages or guiding students toward underserved fields.

The mean age of students (21.27 ± 2.35 years) and the predominance of female students (54.5%) closely mirrored the medical Ain Shams university’s student population and is consistent with other research conducted within the same population. For example, a recent study involving medical students from Ain Shams University reported a similar gender ratio of approximately 44.6% of respondents were males and 55.4% were females, indicating a comparable representation of genders in the student body [23].

This ratio is also aligned with global trends, where female representation in medical schools has surpassed that of males in many countries, aligning with global trends in medical education reflecting an ongoing increase in female enrollment, especially in low- and middle-income nations [24]. These students’ characteristics match findings from several previous studies [18, 25–29].

Almost all (97.8%) students were single, indicating the relatively young age and the demanding nature of medical education, which often postpones family formation [30]. CGPA was included as a variable influencing specialty choice, with 49.9% of students having a CGPA between 3 and 3.5, indicating high academic achievement [13].

The high levels of parental education, particularly among fathers (78.5% reported holding a college or postgraduate degree), align with literature suggesting that students from more educated families are more likely to pursue advanced education and professional careers [31, 32]. Additionally, about half the students reported having at least one family member in the medical field, which has been documented to influence students’ career choices and enhance students’ knowledge of various medical specialties [33].

Across gender, nationality, and academic level, over 94% of students preferred a clinical specialty, whereas only 4.1% were interested in non-clinical specialties. This strong preference toward clinical practice is consistent with prior studies in the Middle East and North African region and internationally [18, 25–29, 34–38], and may be attributed to the perceived greater social impact, prestige, and economic benefits of clinical work. This preference did not differ significantly by gender, nationality, or year of study, indicating a strong and enduring attraction to clinical medicine [39].

This study demonstrated that Internal Medicine and General Surgery were the most common specialties to be chosen, with Obstetrics and Gynecology ranking next. This was like to studies done globally in Saudi Arabia [18, 26, 28, 38], Bahrain [27], India [29], and Germany [20]. This may be due to these specialties being considered core areas of medicine, which students may have encountered or heard about during their early education. Another reasonable explanation might be that early-stage medical students encounter only a limited selection of specialty fields during their studying and have still not gained clinical experience.

The data demonstrated significant associations between specialty selection and both gender and nationality. Male students exhibited preferences for general surgery and internal medicine, while female students demonstrated a predilection for obstetrics and gynecology—a pattern consistent with many studies [25, 40–43]. The findings indicate long-standing gender patterns in specialty choice, reflecting broader geographical tendencies in which cultural norms and expectations of work-life balance often led female students to choose fields that are perceived to offer better work-life balance and dealing with female patients as well.

Nationality significantly influenced specialty selection as well. Egyptian students showed a preference for obstetrics and gynecology and internal medicine, while non-Egyptian students preferred general surgery and cardiology. This difference may reflect differences in perceived opportunities, health care needs, and training and educational availability in their home countries [44, 45].

Passion for the specialty and personal desire were the primary reasons for choosing a field (69.2%), regardless of gender and nationality. This confirms previous findings from Egypt, the Gulf countries, and India indicating that intrinsic motivation is the dominant factor [26, 28, 29, 39, 46, 47]. However, Egyptian students were significantly more influenced by high job opportunities abroad (*p* = 0.049), personal/family time (*p* = 0.028), and less physical exertion (*p* = 0.004). These factors reflect concerns about working conditions and workload issues in the Egyptian healthcare system, which have been previously reported as contributing factors to physician turnover, migration, and burnout [48–52].

Beyond the regional literature, our findings parallel patterns reported in Asian medical schools. An Indian study found that personal growth, professional growth, and personal satisfaction significantly shaped specialty preference among medical students. Similar to our students, Indian respondents ranked personal interest as the dominant determinant; however, financial prospects and workload balance were also prominent concerns, reflecting comparable pressures in rapidly evolving health systems with high patient loads and limited workforce distribution. These parallels highlight that medical students in South Asia and the Middle East often navigate structural constraints that inform their prioritization of lifestyle and future career security [53].

Our findings regarding passion for the specialty and personal desire which drive specialty choice aligns with research in both European and US contexts, where intrinsic motivation is frequently identified as a core determinant. In an European study, personal interest and values around patient care or procedures strongly influenced specialty choice. This suggests that passion and personality fit are pivotal, echoing the findings from the present study [54]. Similarly, in the US, A study found that personal interest and passion for a specialty, more than financial or lifestyle considerations, drive specialty choice among US medical students, underscoring intrinsic motivation as a primary factor [55]. On the other hand, a systematic review found that medical students’ intrinsic interest, rather than external factors such as income or prestige, primarily guided their specialty decisions. This intrinsic motivation is linked to better career satisfaction and persistence in training [56].

Thus, across regions, passion and personal desire remain foundational in specialty choice, emphasizing the universal importance of aligning career paths with personal motivation to foster professional fulfillment and retention [57].

While “personal/family time” is a significant factor in many countries, in Egypt, financial stability, career advancement opportunities, and potential for working abroad are more influential in specialty selection. This reflects socio-economic realities and regional workforce dynamics, consistent with regional studies of medical career motivations. Furthermore, research by ElBeheiry [10] highlights financial push factors such as low remuneration and poor working conditions as key drivers behind physician migration from Egypt, reflecting the interplay between economic pressures and workforce trends.

Our findings reinforce the growing evidence that specialty preferences are shaped not only by individual motivations but also by national and cultural systems that frame what specialties are considered attainable, desirable, or socially valued. Research from the MENA region consistently demonstrates that factors such as societal prestige, anticipated income, family expectations, and exposure to local health-system needs strongly influence medical students’ decisions [58–60]. The differences observed between Egyptian and non-Egyptian students in our study further support this contextual framing, suggesting that variations in home-country training pathways, healthcare priorities, and sociocultural norms continue to shape career aspirations even within a shared educational setting.

Gender differences, however, did not show statistically significant difference. Males were more influenced by the possibility of financial gain, whereas female students while females were more motivated by altruism. This aligns with earlier studies indicating that males often prioritize financial gain and prestige, while females value work-life balance and meaningful patient care [40, 61, 62].

The logistic regression results from our study reinforce a well-documented, multidimensional model of medical students’ specialty choice. Specifically, financial expectations significantly predicted selection of surgical careers, while simultaneously protecting against non-surgical specialties. This is in line with earlier studies indicating that anticipated income remains a powerful motivator for students choosing procedure-intensive or high-prestige specialties [63]. Similarly, work–life balance considerations appeared to steer students away from surgery) and toward non-surgical specialties. This is consistent with the results of a systematic review in which “lifestyle/work–life balance” was among the most frequently cited influences on specialty choice across 54 studies globally [22]. Parallel findings have been presented in regional contexts in a cross-sectional study from Syria [64]. The tendency for students attracted to research opportunities to favor non-surgical fields corresponds with studies indicating that research exposure and academic orientation often draw students toward cognitively focused specialties [65]. Finally, the substantial impact of mentorship on academic career intentions reinforces extensive evidence that role modeling is one of the strongest predictors of long-term engagement in non-clinical medicine [66, 67].

Approximately half of the participating students (51.6%) reported a preference for settling abroad, with this preference significantly higher among non-Egyptians (57.8%). These findings highlight global concerns over physician migration and “brain drain” from developing nations with low to middle incomes, where better training, income, and living conditions are available abroad [8, 62, 68, 69]. The tendency to prefer international work highlights the challenge of brain drain and emphasizes the importance of developing attractive local opportunities for young physicians [70].

The high preference among students for working abroad reflects broader national trends: an Egyptian study showed that 89.4% of Egyptian medical students and young physicians intended to emigrate [11]. According to a different Egyptian study, 85.7% of Egyptian medical students desired to travel overseas after completing their education, mainly to Gulf countries, Europe, and North America, driven by push factors such as low salaries, high workloads, and limited professional recognition [71].

Egyptian students favored the private sector (19.3%) and teaching hospitals (19.3%), while non-Egyptians preferred government hospitals and medical research, probably reflecting the structure, needs, and prestige of these institutions in their home countries. The lack of significant gender variations in these preferences shows that all students, regardless of gender, value international mobility and professional achievements.

The findings of our study should be interpreted in light of larger systemic and policy challenges within the Egyptian health system. First, the national residency allocation mechanism and examination process may restrict medical graduates’ access to certain specialties. This structural barrier can compound personal preference: even when students or interns have strong inclinations toward particular fields, they may not obtain a residency position due to limited slots or central allocation policies [12, 72]. Second, the ongoing physician brain drain significantly affects specialty choice. As reported by Kabbash et al. [11], a large majority (89.4%) of medical students and young physicians surveyed in Egypt intended to emigrate, driven primarily by low remuneration, high workloads, and lack of respect at work. Third, health policy and workforce planning in Egypt must contend with not only the loss of trained physicians but also the inefficiencies in retaining them. The Egyptian public health sector reportedly has a significant gap between the number of registered physicians and those actually practicing in public hospitals. This gap exacerbates shortages in underserved specialties and may further incentivize migration [10].

Finally, given Egypt’s position in a geopolitically significant region and its ongoing socio-political challenges, migration decisions may also be influenced by broader contextual factors, such as regional instability and economic pressures. Although our study did not explicitly examine acceptance rates of emigrating physicians in destination countries, the high intention to emigrate highlights a need for national retention policies. Strengthening working conditions, increasing incentives, and expanding specialty training capacity could help mitigate brain drain and better align specialty choice with national health priorities.

This study has several limitations that should be acknowledged when interpreting the findings. First, its cross-sectional design precludes the ability to capture changes in students’ specialty interests or influencing factors over time; longitudinal approaches would be required to understand how preferences evolve across different stages of medical training. Second, reliance on structured, self-administered questionnaires limits the depth of insight into students’ underlying motivations, as subjective interpretations of items may introduce response bias and restrict the nuanced perspectives that qualitative methods could provide. Additionally, because the study was conducted within a single medical institution in Egypt, the findings may not be fully generalizable to other educational or cultural settings, where differences in healthcare systems, social expectations, and economic conditions may shape specialty preferences differently. Finally, the study examined intended specialty choices rather than actual career outcomes, meaning it cannot determine whether students ultimately pursue or remain in the specialties they indicated. Future research incorporating qualitative methods, multi-institutional samples, and longitudinal follow-up would provide a more comprehensive understanding of specialty selection processes.

## Conclusion

Medical students showed strong interest in clinical specialties, with surgery, internal medicine, and obstetrics and gynecology representing the highest frequently selected fields. Gender and nationality constitute significant factors in shaping students’ career and specialty choices. Their choices were primarily motivated by their passion for the field. However, external factors such as financial aspects and abroad job opportunities also influenced their decisions, particularly for Egyptian students.

### Recommendations

The strong preference for clinical specialties and the pronounced inclination to work abroad underscore the need for targeted interventions for students’ well-being, medical education, specialized career counseling, and workforce planning. Policymakers should address the reasons driving students to seek opportunities abroad, such as improving local training, offering competitive pay, and supporting work-life balance. Additionally, efforts to diversify specialty training and promote non-clinical medicine may help balance workforce distribution and better meet the healthcare system’s needs in Egypt and the broader region.

## Supplementary Information

Supplementary Material 1.

## Data Availability

Upon an appropriate request, data can be obtained from the corresponding author.

## Abbreviations

ASU: Ain Shams University
CGPA: Cumulative Grade Point Average
MENA: Middle East and North Africa
No: Number
P: *P* value
SD: Standard deviation
SPSS: Statistical Package for Social Sciences
X2: Chi-square
