# Supplementary material for: Specialty preferences and influencing factors among undergraduate medical students in Ain Shams University
Source: BMC Med Educ. 2026 Feb 10;26:335. doi: 10.1186/s12909-026-08603-2 (PMC12930952; doi:10.1186/s12909-026-08603-2)
Supplement: Supplementary file 1 — Supplementary Material 1. [file 12909_2026_8603_MOESM1_ESM.docx]

Section I Sociodemographic data

| 1. **Age** | | | |
| --- | --- | --- | --- |
| 1. **Gender** | | | |
| 1. Male | | Female | |
| 1. **Nationality** | | | |
| 1. Egyptian | | | |
| 1. Non-Egyptian | Mention your nationality | | ……………………………. |
| 1. **Marital status** | | | |
| 1. Single | 1. Married | | 1. Divorced |
| 1. **Educational Level** | | |  |
| 1. 1st year | 1. 2nd year | | 1. 3rd year |
| 1. 4th year | 1. 5th year | | 1. Interns (house officer) |
| 1. **Cumulative Grade Point Average (CGPA)** | | |  |
| 1. 2-2.4 | 1. 2.5-2.9 | | 1. 3-3.5 |
| 1. **Father’s education level** | | |  |
| 1. Primary | 1. Intermediate | | 1. Secondary |
| 1. College | 1. Postgraduate studies | | |
| 1. **Mother’s education level** | | |  |
| 1. Primary | 1. Intermediate | | 1. Secondary |
| 1. College | 1. Postgraduate studies | | |
| 1. **Father job** | | | |
| 1. Retired | 1. Medical and health field | | 1. Engineer field |
| 1. Military field | | 1. Education field | |
| 1. A government employee | | 1. Free business\ Private sector | |
| 1. Other (Mention)………………….. | | | |
| 1. **Mother job** | | | |
| 1. Not work | 1. Medical and health field | | 1. Engineer field |
| 1. Military field | | 1. Education field | |
| 1. A government employee | | 1. Free business\ Private sector | |
| 1. Other (Mention)………………….. | | | |
| 1. **Had family member at health care field?** | | | |
| 1. None | 1. Parents | | 1. Siblings |
| d. Other relatives, Mention…………… | | | |

Section II Specialty preference

1. What is the category of your specialty preference?
2. Clinical
3. Academic
4. Not decided
5. What is your specialty preference?
6. Internal medicine
7. General Surgery
8. Family medicine
9. Orthopedics
10. Neurology
11. Obstetrics and Gynecology
12. Emergency Medicine
13. Pediatrics
14. Anesthesia
15. Cardiac Surgery
16. Radiology
17. Clinical pathology
18. ENT
19. Plastic surgery
20. Ophthalmology
21. Dermatology
22. Urology
23. Psychiatry
24. Quality
25. Infection control
26. Oncology
27. Physical medicine
28. Anatomy
29. Physiology
30. Pathology
31. Biochemistry
32. Histology
33. Pharmacology
34. Parasitology
35. Microbiology
36. Community
37. Forensic and toxicology Other:………………………………………………………………………………….

Section III

1. Why do you choose this specialty?............................................................... .............................................................. .........................................................................................................................
2. Which of the following sentences affect your choice of the specialty? (you can choose one or more sentences)

| 1 | Passion for the subject & My personal desire |
| --- | --- |
| 2 | Expected high financial income. |
| 3 | Not requiring much physically exertion. |
| 4 | Can dedicate more time to myself and my family. |
| 5 | My parents or my friends advised me with this specialty. |
| 6 | Innovative field in medicine / great opportunity for scientific research in this specialty. |
| 7 | Influenced by an ideal or a role model or a teacher |
| 8 | High chances of getting foreign scholarships in this field. |
| 9 | High chances of getting jobs in this field abroad. |
| 10 | I prefer to work in teaching and academic institutions only. |
| 11 | Any other reason. |

1. Where do you prefer to work in the future?
2. Government (Ministry of health and Population hospitals)
3. Teaching hospital
4. Armed forces
5. Private sector
6. Medical research
7. Settle abroad
8. Settle in rural areas
9. Teaching
10. Other (mention) ……………………….
